# Exploring the potential mechanism of emetine against coronavirus disease 2019 combined with lung adenocarcinoma: bioinformatics and molecular simulation analyses

**DOI:** 10.1186/s12885-022-09763-2

**Published:** 2022-06-22

**Authors:** Kun Zhang, Ke Wang, Chaoguo Zhang, Xiuli Teng, Dan Li, Mingwei Chen

**Affiliations:** Department of Respiratory and Critical Care Medicine, First Affiliated Hospital of Xi’an Jiaotong University, No. 277 Yanta West Road, Xi’an, 710061 Shaanxi Province China

**Keywords:** COVID-19, Lung adenocarcinoma, Bioinformatics analyses, Molecular docking, Emetine, Molecular dynamics

## Abstract

**Background:**

Patients with lung adenocarcinoma (LUAD) may be more predisposed to coronavirus disease 2019 (COVID-19) and have a poorer prognosis. Currently, there is still a lack of effective anti-LUAD/COVID-19 drugs. Thus, this study aimed to screen for an effective anti-LUAD/COVID-19 drug and explore the potential mechanisms.

**Methods:**

Firstly, we performed differentially expressed gene (DEG) analysis on LUAD transcriptome profiling data in The Cancer Genome Atlas (TCGA), where intersections with COVID-19-related genes were screened out. Then, we conducted Cox proportional hazards analyses on these LUAD/COVID-19 DEGs to construct a risk score. Next, LUAD/COVID-19 DEGs were uploaded on Connectivity Map to obtain drugs for anti-LUAD/COVID-19. Finally, we used network pharmacology, molecular docking, and molecular dynamics (MD) simulation to explore the drug’s therapeutic targets and potential mechanisms for anti-LUAD/COVID-19.

**Results:**

We identified 230 LUAD/COVID-19 DEGs and constructed a risk score containing 7 genes (*BTK*, *CCL20*, *FURIN*, *LDHA*, *TRPA1*, *ZIC5*, and *SDK1*) that could classify LUAD patients into two risk groups. Then, we screened emetine as an effective drug for anti-LUAD/COVID-19. Network pharmacology analyses identified 6 potential targets (*IL6*, *DPP4*, *MIF*, *PRF1*, *SERPING1*, and *SLC6A4*) for emetine in anti-LUAD/COVID-19. Molecular docking and MD simulation analyses showed that emetine exhibited excellent binding capacities to DDP4 and the main protease (Mpro) of severe acute respiratory syndrome coronavirus 2 (SARS-CoV-2).

**Conclusions:**

This study found that emetine may inhibit the entry and replication of SARS-CoV-2 and enhance tumor immunity by bounding to DDP4 and Mpro.

**Supplementary Information:**

The online version contains supplementary material available at 10.1186/s12885-022-09763-2.

## Background

Coronavirus disease 19 (COVID-19) is caused by severe acute respiratory syndrome coronavirus 2 (SARS-CoV-2), a novel virus that can be transmitted from one person to another [1]. By January 3, 2022, more than 290 million individuals worldwide have been diagnosed with COVID-19, and more than 5.4 million of these patients have died [2]. Although some drugs have been used against COVID-19, such as paxlovid, molnupiravir [3], and monoclonal antibodies against SARS-CoV-2 [4], there is still a lack of effective specific anti-COVID-19 drugs [5], especially when COVID-19 is combined with some other diseases. Therefore, we need to screen and validate potential bioactive drugs against COVID-19. Additionally, lung cancer patients may be at an increased risk of contracting SARS-CoV2 than individuals without cancer, and these cancer patients with COVID-19 show a worse prognosis [6]. Generally, lung cancer patients exhibit multiple immune abnormalities [7], which would affect the efficacy of anti-COVID-19 treatment. Lung cancer ranks as the second most prevalent malignancy worldwide in terms of incidence and mortality, in which lung adenocarcinoma (LUAD) is the subtype that accounts for approximately 40 percent [8–10]. Considering that the LUAD patients are more predisposed to COVID-19 and have a worse prognosis, we should screen for effective therapeutic drugs.

Emetine is a plant-derived alkaloid that was earlier used as an antiprotozoal and emetic agent [11]. Recent studies have shown that emetine exhibited antitumor effects in various cancers through various pathways. For example, emetine sensitizes ovarian and bladder cancer cells to cisplatin [12, 13], and emetine exhibits anticancer activity in breast cancer and non-small cell lung cancer (NSCLC) cells [14, 15]. In addition, emetine can decrease contracting Zika and Ebola viruses by suppressing the replication and invasion of viruses [16]. Recently, some studies on COVID-19 have suggested that emetine may inhibit SARS-CoV-2 by binding to its main protease (Mpro) [17] and papain-like protease (PLpro) [18], while others have suggested that emetine exerts anti-COVID-19 effects in other ways [19–21]. The SARS-CoV-2 genome encodes two proteases: PLpro and Mpro, and they are potential drug targets. PLpro is involved in forming the coronavirus replicase complex and viral RNA replication and transcription [22]. At the same time, Mpro is required to process the polypeptides produced by viral RNA translation [23]. Given the above controversial findings, emetine’s pharmacological targets and potential mechanisms against LUAD/COVID-19 remain further investigated.

**Fig. 1:**
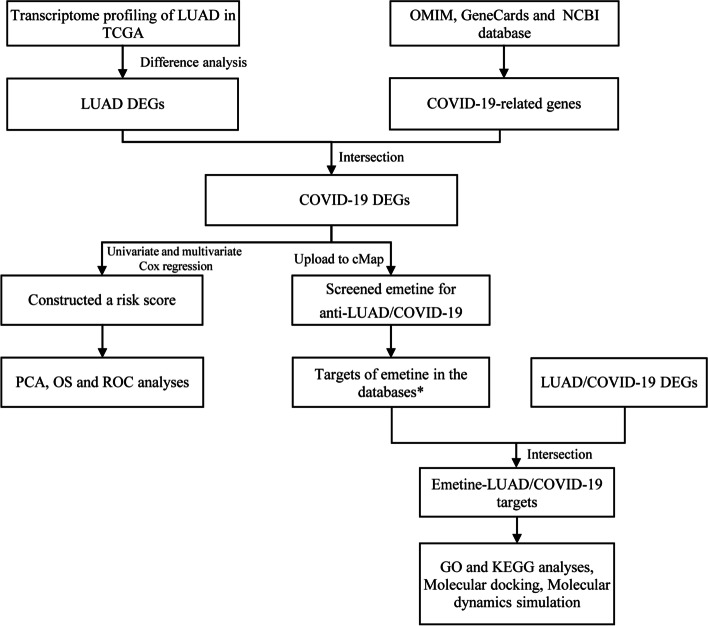
Flow chart of the study. *HERB, TargetNet, Batman, PharmMapper, PubChem, Similarity ensemble approach (SEA), and ChEMBL

The present study aimed to screen for effective drugs to treat LUAD/COVID-19 and investigate the potential mechanisms. Using bioinformatics analyses, we firstly built a risk score according to LUAD/COVID-19 differential expression genes (DEGs) to predict the prognosis of LUAD patients in The Cancer Genome Atlas (TCGA), and we screened emetine as a possible anti-LUAD/COVID-19 drug. Then, we identified the emetine’s targets via network pharmacology and explored the potential mechanisms of emetine against LUAD/COVID-19 by molecular docking and molecular dynamics (MD) simulation. The flow chart for this research is shown in Fig. 1.

## Methods

### Identification of LUAD/COVID-19 DEGs

We downloaded the transcriptome profiling data of LUAD from the TCGA database (October 1, 2021) [24]. The R package “limma” was utilized to conduct the difference analysis of the LUAD transcriptome profiling data, where the filtering conditions were | log (fold change) | > 1 and false discovery rate < 0.05. Then, we downloaded and screened COVID-19-related genes from the OMIM database, GeneCards database (relevance score > 1), and the NCBI gene database. Finally, we obtained the LUAD/COVID-19 DEGs by taking the intersection of the COVID-19-related genes with the DEGs of LUAD [25].

### Clinical prognostic analysis of LUAD/COVID-19 DEGs

We conducted the univariate Cox regression analysis to assess the correlation of each LUAD/COVID-19 gene with the survival status of LUAD sufferers. *P*-value < 0.01 was considered related to the patients’ prognosis in LUAD. Then, we performed the multivariate Cox regression analysis on the prognosis-related genes obtained above and constructed a risk score. The risk score formula is risk score = ${\sum}_i^7\mathrm{X}i\ast Yi$ (*X*: gene expression level; *Y*: coefficient). According to the risk score at the median, we classified LUAD patients as high and low risk. Moreover, we performed the Kaplan-Meier analysis to compare the overall survival time of the two subgroups. Also, we performed a principal component analysis utilizing the “prcomp” function in the R programming language. The ROC curve analyses were performed utilizing the R packages, including “survival,” “survminer,” and “timeROC” [26]. Additionally, we compared the association of each gene in the risk score with distinct clinical characteristics, including age, gender, stage, T, N, and M.

### Screening of anti-LUAD/COVID-19 drugs

We used the “Perl” language to convert the up- and downregulated LUAD/COVID-19 DEGs into the corresponding probe IDs, and we uploaded the obtained probe IDs to the Connectivity Map (cMap) website [27]. By querying in cMap, drugs with similar or opposite expressed gene patterns were available. Then, the *P*-value < 0.05 and enrichment value < 0 were used to screen anti-LUAD/COVID-19 drugs.

### Target network construction for emetine and LUAD/COVID-19

We screened and collected possible human targets of emetine from available online tools such as HERB, TargetNet, Batman, PharmMapper, PubChem, Similarity ensemble approach, and ChEMBL. The genes corresponding to targets of emetine were compared with LUAD/COVID-19 DEGs, and the protein-protein interaction network of intersecting genes was obtained in the STRING database (version 11.5). The network analyzer setting in the Cytoscape software (version 3.7.2) was utilized to analyze the topological parameters.

### Functional enrichment analysis and network visualization

Gene Ontology (GO) and Kyoto Encyclopedia of Genes and Genomes (KEGG) analyses and visualization of the intersecting genes of emetine and LUAD/COVID-19 were performed using the R package “ClusterProfiler.” The outputs of the enrichment analyses were presented as bar plots. In addition, the Cytoscape software was utilized to construct the emetine-targets-GO function-KEGG pathway-LAUD/COVID-19 network.

### Redocking

To examine the reliability of autodock vina’s prediction of drug and target binding patterns, we used the redocking method to validate the molecular docking method and parameters. We separated the native ligand and receptor in the co-crystal structure, preprocessed them using AutoDockTools, and finally performed molecular docking. We used the “align” command in PyMOL to calculate the RMSD of the ligand conformation predicted by autodock vina with the ligand conformation in the co-crystal structure. The redocking protocol was considered valid for RMSD < 2 Å [28, 29].

### Molecular docking

To assess whether emetine can bind to SARS-CoV-2 Mpro, we used a molecular docking approach to predict the potential binding sites of emetine. From the PubChem database, we downloaded the molecular structure of emetine [30]. The protein structures of Mpro and DDP4 were obtained from the PDB database [31]. The PDB ID of Mpro is 6LZE [32, 33], and the PDB ID of DDP4 is 4PNZ [34, 35]. We used the ChemBio3D Ultra 2014 wizard software to add hydrogen atoms and minimize energy to small-molecule structures. Before molecular docking, the ligand and receptor were prepared using AutoDockTools-1.5.7, which mainly involved removing water molecules from the receptor, adding hydrogen atoms, and generating coordinate files. At the same time, the ligands were detected root, chose torsions, and generated coordinate files [36, 37]. Then, we generated the grid box based on the receptor active site, and the size and coordinates of the grid box are as follows. The coordinates *x* = − 16.24, *y* = 21.64, and *z* = 68.796 were considered the center of the grid at the active site of Mpro with dimensions of 30.0, 38.0, and 38.0, respectively. This pocket of Mpro includes the active site confirmed by previous work, which is occupied by amino acids ASN142, CYS44, CYS145, GLY143, GLU166, GLN189, GLN192, HIS163, HIS164, HIS172, HIS41, LEU27, LEU141, MET49, PRO52, PHE140, PHE181, SER139, THR24, THR26, and THR190 [38, 39]. The coordinates *x* = 44.995, *y* = 52.024, and *z* = 40.079 were considered the center of the grid at the active site of DDP4 with dimensions of 52.0, 54.0, and 52.0, respectively. This pocket of DDP4 includes amino acid residues from the active site of DDP4, including HIS740, SER630, ARG125, GLU205, GLU206, TYR547, PHE347, SER209, TYR585, and ARG348 [34, 40]. The other parameters were set as follows: exhaustiveness = 10, models = 20, and energy range = 4. A root mean square deviation (RMSD) cluster analysis, which used an RMSD-tolerance of 2.0 Å, was performed using the ligand atoms only. The Autodock Vina program was utilized to accomplish molecular docking [41]. We used PyMOL to visualize the results and show the receptor residues and the hydrogen bonds between protein ligands and receptors [42].

### MD simulation

We performed 100 ns MD simulations of the protein-ligand complexes obtained by molecular docking and compared them with the corresponding co-crystal inhibitors in each case [43]. This study used the GROMACS software (2020.6-MODIFIED version) running on Linux operating system [44]. The results were visualized using the QTGrace software [45]. The AMBER99SB-ILDN force field was used to generate the protein topology. AmberTools and ACPYPE were used to create GAFF force fields and parameters for the ligands, and the AM1-bcc charges were calculated using the antechamber program [46]. The TIP 3-point solvent model was used to solvate each system, then neutralized with appropriate amounts of Na and Cl+-. Energy minimization was used to minimize the overall potential energy of the protein and ligand [47]. The energy of each system was minimized by using the steepest descent minimization algorithm that stopped minimization at > 50,000 steps and a maximum force <10.0 kJ/mol. Each system underwent a 100-ps NVT equilibration and a 200-ps NPT equilibration process. We equilibrated the system by NPT at 1 bar pressure and 300K temperature [48, 49]. Particle mesh Ewald was used to treat long-range electrostatics, and the Fourier transform grid spacing was set to 0.16. However, solvent molecules were allowed to move freely to establish solvent equilibrium in the system.

MD simulations were performed for each equilibrium system for 100 ns with a time step of 2 fs. Structural coordinates were saved every 10 ps. Various parameters such as RMSD and root mean square fluctuation (RMSF) were calculated for 100 ns MD [50]. To evaluate the stability of protein-ligand complexes in 100 ns MD simulations, we examined the formation of hydrogen bonds during 100 ns and generated the hydrogen bond (H-bond) monitoring reports [47]. In addition, to quantify the strength of the interaction between the ligands and the proteins, we calculated the non-bonded interaction energy between the proteins and the ligands using GROMACS [51]. The cutoff values of short-range electrostatic interactions and Van Der Waals (Vdw) were 1.2 nm.

## Results

### Identification of LUAD/COVID-19 DEGs

**Fig. 2:**
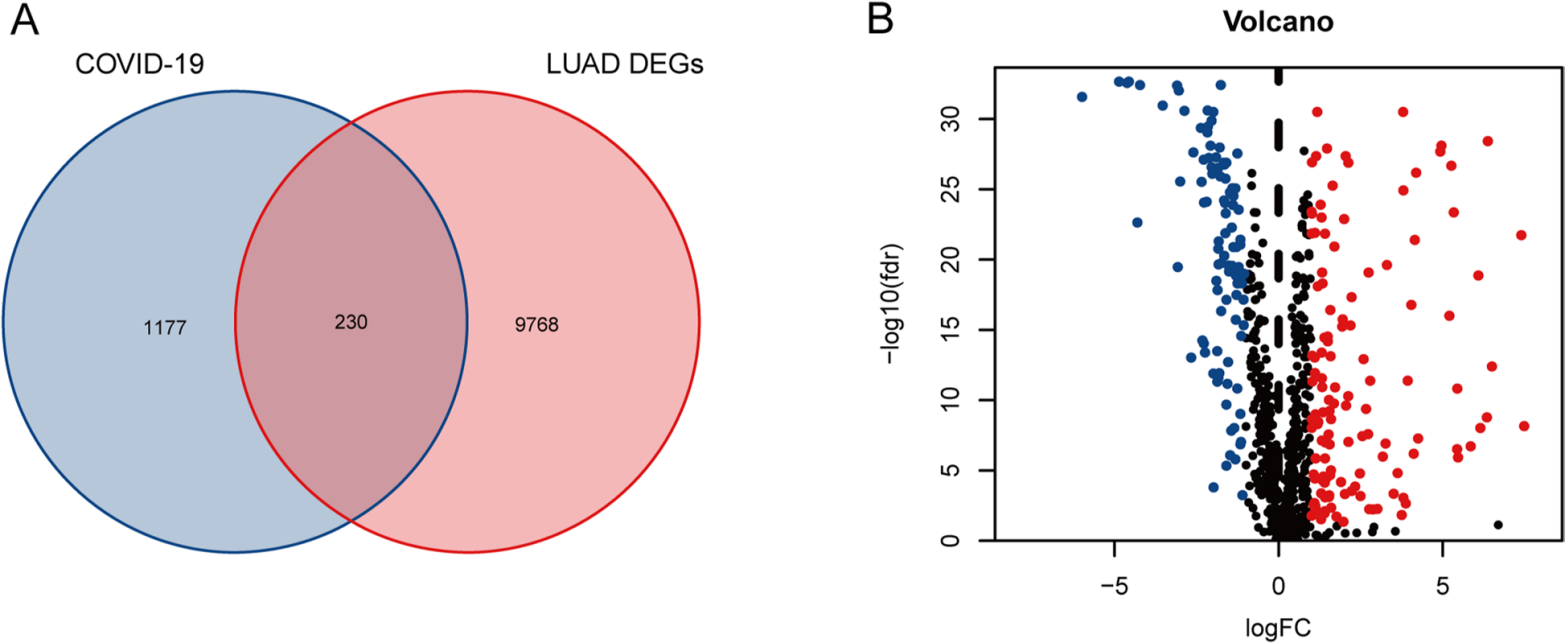
Identification of LUAD/COVID-19 DEGs. **A** The intersection of LUAD DEGs and COVID-19-related genes. **B** Volcano plot of intersecting DEGs

We identified 9998 DEGs from the LUAD transcriptome profiling in the TCGA database (Fig. 2A). Then, we collected 1407 COVID-19-related genes from the databases (Fig. 2A, Additional file 1). Finally, 230 intersecting genes were found in the COVID-19 and LUAD gene clusters (Fig. 2A). Moreover, 130 genes out of 230 DEGs were highly expressed, while the other 100 were lowly expressed in tumor tissues (Fig. 2B, Additional file 2).

### Clinical prognostic analysis of LUAD/COVID-19 DEGs

**Fig. 3:**
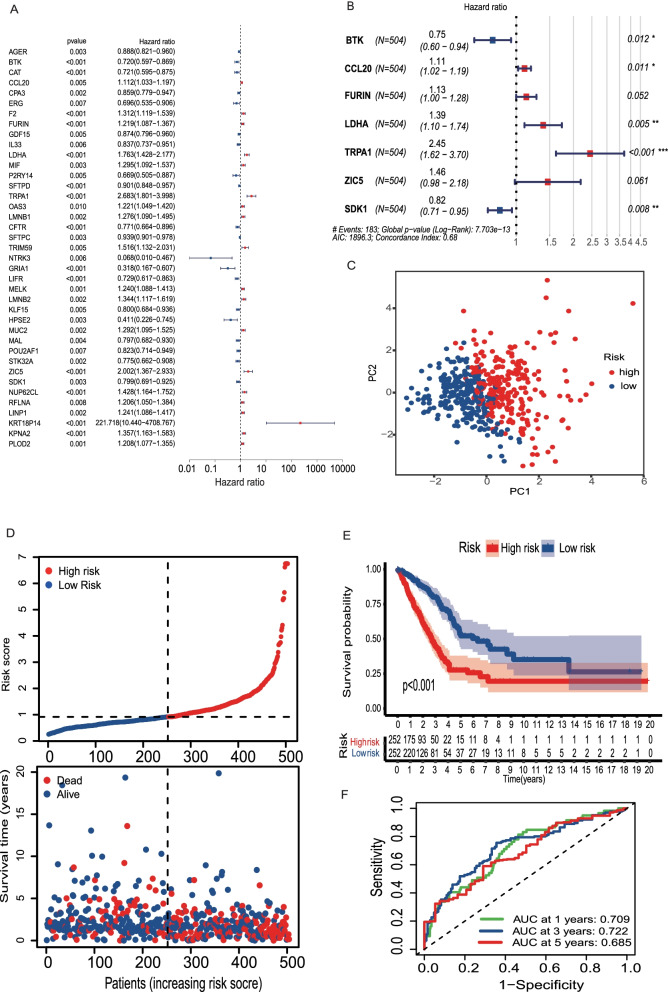
Construction of a risk score. **A** Univariate Cox analysis of LUAD/COVID-19 DEGs. **B** Multivariate Cox analysis of LUAD/COVID-19 DEGs. **C** PCA plot of LUAD patients. **D** The median risk score and the distributions of survival status. **E** Kaplan-Meier analysis for the two different risk groups. **F** ROC of the risk score

**Fig. 4:**
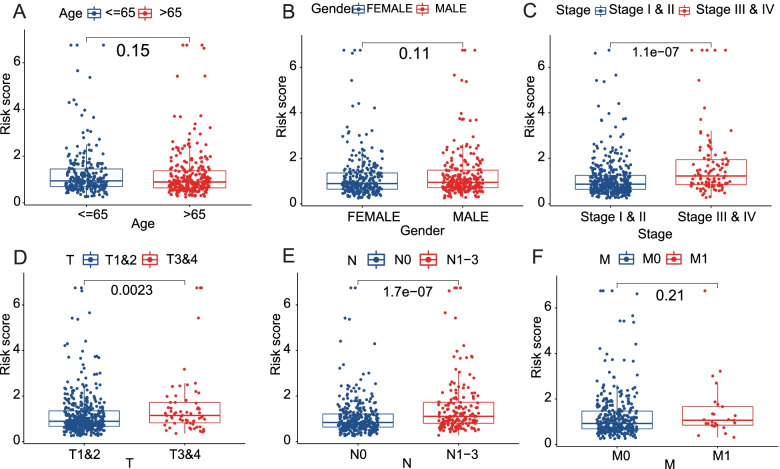
Clinical prognostic analysis of the risk score. **A**–**F** The relationship of risk scores and age, gender, stage, T, N, and M in LUAD patients

**Fig. 5:**
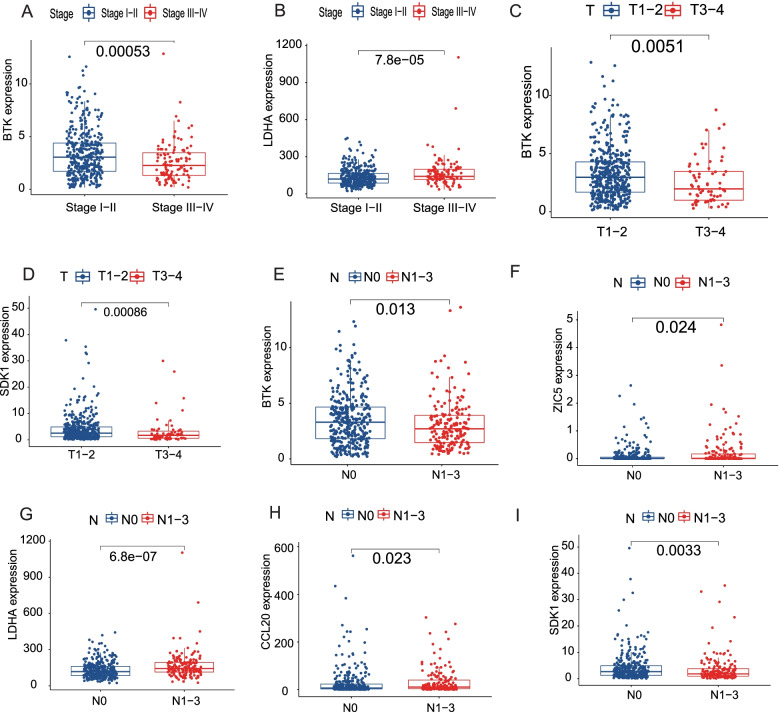
Clinical prognostic analysis of genes in the risk score. **A**, **B** The relationship of BTK and LDHA with stage in LUAD patients. **C**, **D** The relationship of *BTK* and *SDK1* with T in LUAD patients. **E**–**I** The relationship of *BTK*, *ZIC5*, *LDHA*, *CCL20*, and *SDK1* with N in LUAD patients

For clinical prognostic analysis, we collected clinical information on LUAD patients in TCGA (Table 1), of whom 504 patients had complete survival times and gene expression profiles. Then, the Cox proportional hazards analyses were performed on the 230 LUAD/COVID-19 DEGs to explore the correlation between these genes and the clinical prognosis of the LUAD patients. The results of the univariate Cox regression analysis showed that 39 LUAD/COVID-19 DEGs were significantly associated with patient prognosis (*P* < 0.01) (Fig. 3A, Additional file 3). Then, the multivariate Cox regression analysis was conducted on the 39 prognosis-related DEGs, and we constructed a risk score consisting of 7 genes, including *BTK*, *CCL20*, *FURIN*, *LDHA*, *TRPA1*, *ZIC5*, and *SDK1* (Fig. 3B, Table 2). The principal component analysis confirmed that LUAD patients were classified into two distinct risk subgroups (Fig. 3C). Furthermore, compared with the group at low risk, mortality was higher and survival time was shorter in the high-risk group (Fig. 3D, E, *P* < 0.001). Meanwhile, we assessed the specificity and sensitivity of the risk score using ROC curve analyses, which exhibited the AUC of 0.709 at 1 year, 0.722 at 3 years, and 0.685 at 5 years (Fig. 3F). We compared the risk scores of patients with distinct clinical features. The results demonstrated that the risk scores for patients in stages III–IV are higher than those for patients in stages I–II, further confirmed by T and N stages (Fig. 4). Furthermore, the analyses of these 7 gene expression levels between different clinical features suggested that patients with advanced-stage LUAD had lower expression of *BTK* and *SDK1* and higher expression of *CCL20*, *LDHA*, and *ZIC5*. Moreover, the expressed levels of *BTK*, *SDK1*, *CCL20*, *LDHA*, and *ZIC5* correlated with regional lymph node metastasis (Fig. 5).

**Table 1:** Baseline characteristics of LUAD patients in TCGA

	All (*n* = 522)	Alive (*n* = 334)	Dead (*n* = 188)	*P*_overall_
Age, years				0.678
> 65, *n* (%)	262 (50.2%)	163 (48.8%)	99 (52.7%)	
≤ 65, *n* (%)	241 (46.2%)	159 (47.6%)	82 (43.6%)	
Unknown, *n* (%)	19 (3.64%)	12 (3.59%)	7 (3.72%)	
Gender				0.668
Female, *n* (%)	280 (53.6%)	182 (54.5%)	98 (52.1%)	
Male, *n* (%)	242 (46.4%)	152 (45.5%)	90 (47.9%)	
Stage				< 0.001
Stages I–II, *n* (%)	403 (77.2%)	280 (83.8%)	123 (65.4%)	
Stages III–IV, *n* (%)	111 (21.3%)	48 (14.4%)	63 (33.5%)	
Unknown, *n* (%)	8 (1.53%)	6 (1.80%)	2 (1.06%)	
T				0.005
T1–2, *n* (%)	453 (86.8%)	301 (90.1%)	152 (80.9%)	
T3–4, *n* (%)	66 (12.6%)	32 (9.58%)	34 (18.1%)	
Unknown, *n* (%)	3 (0.57%)	1 (0.30%)	2 (1.06%)	
N				< 0.001
N0, *n* (%)	335 (64.2%)	245 (73.4%)	90 (47.9%)	
N1-3, *n* (%)	175 (33.5%)	81 (24.3%)	94 (50.0%)	
Unknown, *n* (%)	12 (2.30%)	8 (2.40%)	4 (2.13%)	
M				0.003
M0, *n* (%)	353 (67.6%)	219 (65.6%)	134 (71.3%)	
M1, *n* (%)	25 (4.79%)	10 (2.99%)	15 (7.98%)	
Unknown, *n* (%)	144 (27.6%)	105 (31.4%)	39 (20.7%)	

*LUAD* lung adenocarcinoma, *TCGA* The Cancer Genome Atlas

**Table 2:** Multivariate Cox proportional hazards regression analysis

Symbol	coef	HR	HR.95L	HR.95H	*P*-value
BTK	− 0.28547	0.751663	0.601526	0.939275	0.012038
CCL20	0.100563	1.105793	1.023249	1.194996	0.011066
FURIN	0.121873	1.12961	0.998889	1.277439	0.052107
LDHA	0.326232	1.385737	1.104652	1.738345	0.004795
TRPA1	0.894946	2.447203	1.61896	3.699167	2.18E-05
ZIC5	0.381837	1.464974	0.983117	2.183004	0.060615
SDK1	-0.19845	0.820004	0.708347	0.949261	0.007879

### Screening of anti-LUAD/COVID-19 drugs

We uploaded 130 upregulated and 100 downregulated LUAD/COVID-19 DEGs to cMap for querying, and drugs with similar or opposite gene expression patterns could be obtained. The obtained drugs were screened according to the screening criteria, resulting in 19 potential anti-LUAD/COVID-19 drugs (Table 3). Then, we chose emetine as a promising medication against LUAD/COVID-19 for further study.

**Table 3:** Screening for anti-LUAD/COVID-19 drugs based on cMap

Rank	cMap name	Mean	Number	Enrichment	*P*-value
1	Puromycin	− 0.729	4	− 0.959	0
2	Emetine	− 0.522	4	− 0.83	0.00151
3	Glycocholic acid	− 0.506	4	− 0.815	0.00211
4	Chicago Sky Blue 6B	− 0.337	4	− 0.797	0.00336
5	Nitrofural	− 0.297	4	− 0.781	0.00462
6	Cephaeline	− 0.36	5	− 0.758	0.00154
7	Pepstatin	− 0.326	4	− 0.711	0.0141
8	Cinoxacin	− 0.569	4	− 0.711	0.01414
9	4-Hydroxyphenazone	− 0.356	5	− 0.702	0.00511
10	Oxymetazoline	− 0.502	4	− 0.702	0.01647
11	Primidone	− 0.347	4	− 0.671	0.02626
12	Lycorine	− 0.34	5	− 0.669	0.00917
13	Parthenolide	− 0.548	4	− 0.661	0.02998
14	Sulindac	− 0.437	7	− 0.654	0.00178
15	Citalopram	− 0.318	4	− 0.642	0.03929
16	*N*-acetylmuramic acid	− 0.322	4	− 0.64	0.03995
17	Tropine	− 0.357	4	− 0.639	0.04084
18	Ciclosporin	− 0.29	6	− 0.594	0.01577
19	Benzamil	− 0.302	6	− 0.542	0.03665

### Target network construction for emetine and LUAD/COVID-19

**Fig. 6:**
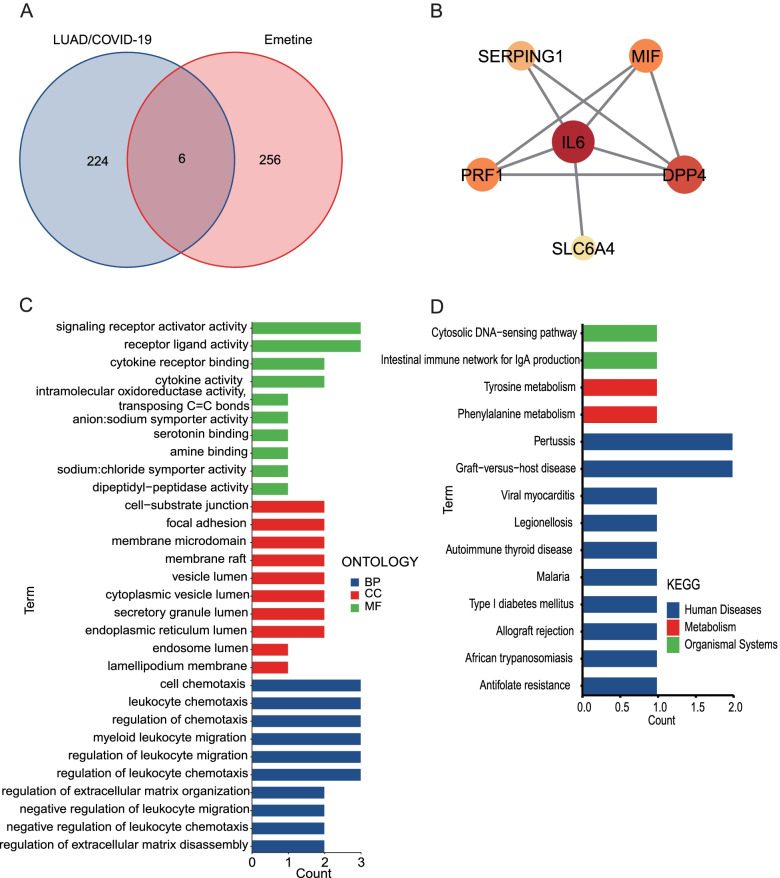
Functional identification of the targets of emetine against LUAD/COVID-19. **A** The intersection of emetine’s targets and LUAD/COVID-19 DEGs. **B** PPI network of SLC6A4, MIF, DPP4, PRF1, SERPING1, and IL6. **C** Bar plot of GO enrichment analysis of intersecting genes. **D** Bar plot of KEGG enrichment analysis of intersecting genes [52–54]

We screened and collected 262 possible human targets for emetine from online tools such as HERB, TargetNet, Batman, PharmMapper, PubChem, Similarity ensemble approach, and ChEMBL. The genes corresponding to these targets were compared with 230 LUAD/COVID-19 DEGs, and 6 intersecting genes were obtained (Fig. 6A), including *SLC6A4*, *MIF*, *DPP4*, *PRF1*, *SERPING1*, and *IL6*. In addition, we visualized the interaction of these intersecting targets and performed a network topology analysis (Fig. 6B).

### Functional enrichment analysis of intersecting targets

Subsequently, we conducted functional enrichment analysis on *SLC6A4*, *MIF*, *DPP4*, *PRF1*, *SERPING1*, and *IL6*. The results of GO revealed that the top biological process terms were regulation of chemotaxis, leukocyte chemotaxis, and cell chemotaxis, and the top cellular component terms were the cell-substrate junction, focal adhesion, and vesicle lumen. Furthermore, the top molecular function terms were signaling receptor activator activity and receptor-ligand activity (Fig. 6C, Additional file 4). Also, the results of the KEGG pathway analysis showed that the 6 genes were significantly related to the cytosolic DNA-sensing pathway, the intestinal immune network for IgA production, tyrosine metabolism, phenylalanine metabolism, pertussis, graft-versus-host disease, viral myocarditis, legionellosis, autoimmune thyroid disease, malaria, type I diabetes mellitus, allograft rejection, African trypanosomiasis and antifolate resistance (Fig. 6D, Additional file 5). In addition, we used the Cytoscape software to map the network of emetine against LUAD/COVID-19 targets and the interactions of related pathways (Fig. 10).

### Redocking

**Fig. 7:**
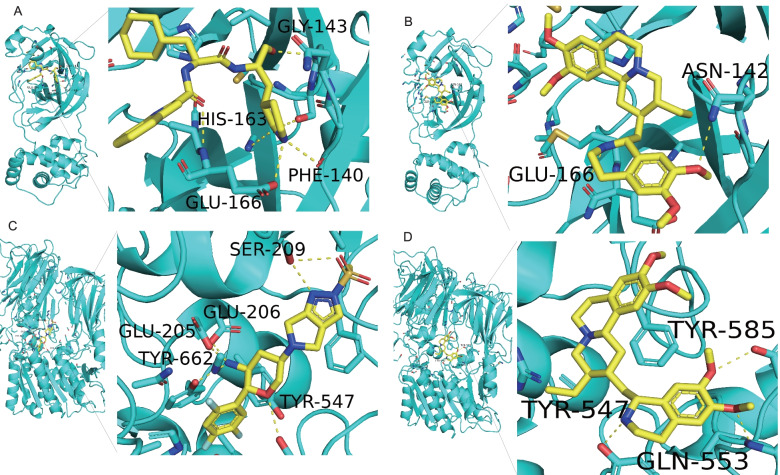
Molecular docking of emetine with SARS-CoV-2 Mpro and DDP4. **A** The binding site of the original ligand (inhibitor 11a) to Mpro. **B** Hydrogen bonds formed between emetine and Mpro on GLU-166 and ASN-142. **C** The binding site of the original ligand (omarigliptin) to DDP4. **D** Hydrogen bonds formed between emetine and DDP4 on TYR547, TYR585, and GLN553

Initially, we performed redocking of the native ligand and protein in co-crystal to validate the molecular docking scheme. The redocking results showed that inhibitor 11a had an RMSD value of 0.712 Å after redocking on Mpro (Table 4). Moreover, inhibitor 11a formed 6 hydrogen bonds with amino acid residues of Mpro, including HIS-163, PHE-140, GLY-143, and GLU-166 (affinity − 8.3 kcal/mol, Fig. 7A, Table 4). Omarigliptin had an RMSD value of 0.790 Å after redocking on DDP4 (Table 4), and omarigliptin formed 6 hydrogen bonds with the amino acid residues of DDP4, including TYR-662, TYR-547, GLU-205, GLU-206, and SER209 (affinity − 9.2 kcal/mol, Fig. 7C, Table 4).

**Table 4:** RMSD values after redocking of two ligands on Mpro and DDP4

Protein target	PDB code	RMSD(A)	Native ligand	Affinity (kcal/mol)	Hydrogen bonds	Interacting residues
Mpro	6LZE	0.712	Inhibitor 11a	− 8.3	6	HIS-163, PHE-140, GLY-143, and GLU-166
DDP4	4PNZ	0.790	Omarigliptin	− 9.2	6	TYR-662, TYR-547, GLU-205, GLU-206, and SER209

### Molecular docking

To further explore the potential efficacy of emetine against LUAD/COVID-19, we performed molecular docking analyses of emetine with Mpro and candidate targets, and emetine formed 2 hydrogen bonds with amino acid residues of Mpro, including GLU-166 and ASN-142 (Fig. 7B), which indicated that emetine had good binding activity to Mpro (affinity − 7.8 kcal/mol, Table 5). In addition, we evaluated the potential bindings of emetine with the LUAD/COVID-19 targets (SLC6A4, MIF, DPP4, PRF1, SERPING1, and IL6). The molecular docking suggested that emetine may bind with DDP4 and MIF, whereby emetine had the best binding activity to DDP4. Emetine formed 3 hydrogen bonds with the amino acid residues of DDP4, including TYR547, TYR585, and GLN553, where TYR547 was also the hydrogen bond formed by the native ligand binding to DDP4 (affinity − 8.7 kcal/mol, Fig. 7D, Table 5). This result indicated that emetine had an excellent binding ability to DDP4. In addition, the binding pattern and binding capacity of emetine and MIF are shown in Additional file 6.

**Table 5:** Assessment of molecular docking of emetine with SARS-CoV-2 Mpro and DDP4

	Affinity (kcal/mol)	Hydrogen bonds	Interacting residues
Mpro			
Emetine	− 7.8	2	GLU-166 and ASN-142
DDP4			
Emetine	− 8.7	3	TYR547, TYR585, and GLN553

### MD simulation

#### RMSD analysis

**Fig. 8:**
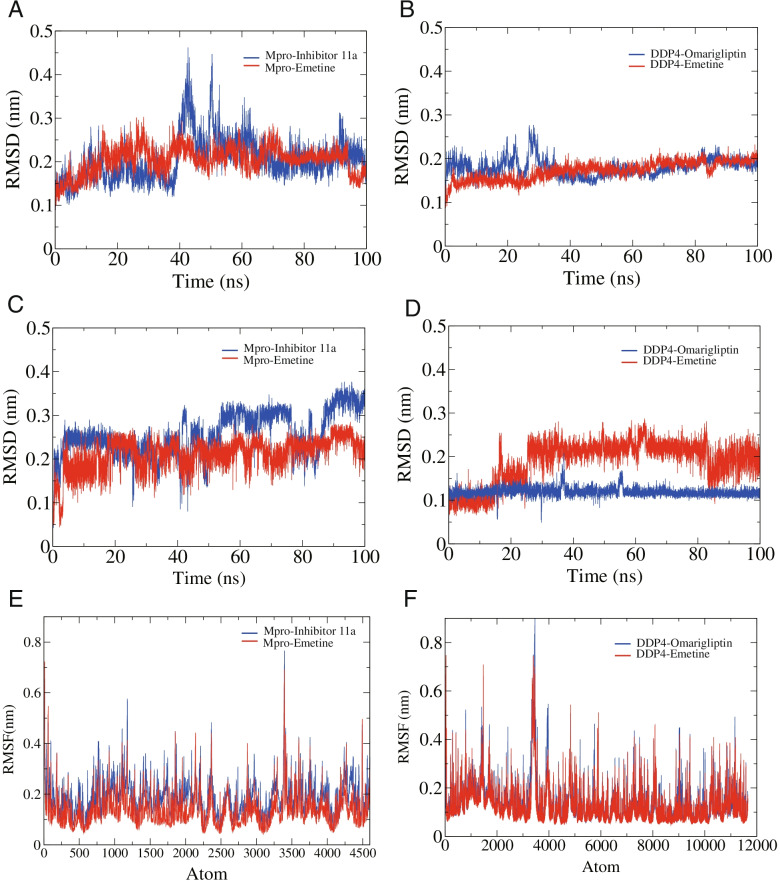
RMSD and RMSF of the SARS-CoV-2 Mpro complexes and DDP4 complexes. **A** The RMSD of Mpro. **B** The RMSD of DDP4. **C** The RMSD of inhibitor 11a and emetine. **D** The RMSD of omarigliptin and emetine. **E** The RMSF of Mpro. **F** The RMSF of DDP4

To study the conformational stability of the complexes of proteins and ligands obtained by molecular docking, we performed the 100-ns MD simulations. RMSD reflects the degree of the positional change of the molecular structure over time. MD simulations showed that in the Mpro-inhibitor 11a complex system, Mpro exhibited good stability within 40 ns with an average RMSD < 0.2 nm. During 40–60 ns, the RMSD value fluctuated with a maximum value of 0.45 nm, while during 60–100 ns, Mpro stabilized again, fluctuating between 0.14 and 0.35 nm with an average RMSD of 0.21 nm (Fig. 8A). In the MD simulation of the Mpro-emetine complex system, Mpro showed good stability, and the RMSD was in the range of 0.1–0.3 nm with an average value of 0.2 nm during the whole 100 ns (Fig. 8A). Figure 8B showed the RMSD fluctuations of DDP4 in the complexes formed with native ligand (omarigliptin) and emetine, respectively. Although in the DDP4-omarigliptin system, DDP4 showed large fluctuations within 40 ns, with a maximum RMSD of 0.28 nm, it was stable from 40 to 100 ns, fluctuating from 0.14 to 0.24 nm with an average value of 0.18 nm (Fig. 8B). In the DDP4-emetine system, the RMSD of DDP4 was in the range of 0.08–0.23 nm with an average value of 0.17 nm (Fig. 8B).

Figure 8C, D showed the conformational changes of the ligands in each of the four complex systems. As shown in Fig. 8C, in the complex system formed with Mpro, the RMSD of inhibitor 11a was in the range of 0.04–0.38 nm, where it fluctuated in the range of 0.04–0.32 nm within 50 ns, 0.14–0.35 nm from 50 to 90 ns, and 0.27–0.38 nm from 90 to 100 ns (Fig. 8C). The RMSD of emetine was in the range of 0.04–0.28 nm, with a mean value of 0.21 nm, and the overall equilibrium was achieved after 5 ns (Fig. 8C). In the complex system formed with DDP4, the RMSD of omarigliptin was in equilibrium in the range of 0.02–0.21 nm at an early stage and was maintained until 100 ns (Fig. 8D), and the RMSD of emetine was in equilibrium at 30–100 ns, and the RMSD was in the range of 0.09–0.29 nm with an average value of 0.21 nm (Fig. 8D).

#### RMSF and radius of gyration analyses

RMSF can show the degree of free movement of atoms and flexibility of amino acid residues in the receptor molecule. As shown in Fig. 8E, F, the maximum values of RMSF for Mpro and DDP4 were around 0.80 nm and 0.9 nm, while most residues fluctuated in the RMSF range of 0.04 nm, indicating that some residues of both receptors possessed great flexibility. Overall, the Mpro backbone atoms had low oscillations, which to some extent described the stable behavior of the protein-ligand complexes. Moreover, compared with native ligands, emetine showed similar fluctuations of amino acid residues when binding Mpro and DDP4, indicating the potential of emetine binding activity. In addition, we analyzed the radii of gyration of Mpro and DDP4, which can reflect the compactness of the protein structure. In addition, we analyzed the radii of gyration of Mpro and DDP4, which can reflect the compactness of the protein structure. Compared with native ligands, the radii of gyration of emetine when bound to Mpro and DDP4 were comparable and remained at relatively stable values, which indicated the stability of the protein folding conformation (Additional file 7).

#### H-bond monitoring

**Fig. 9:**
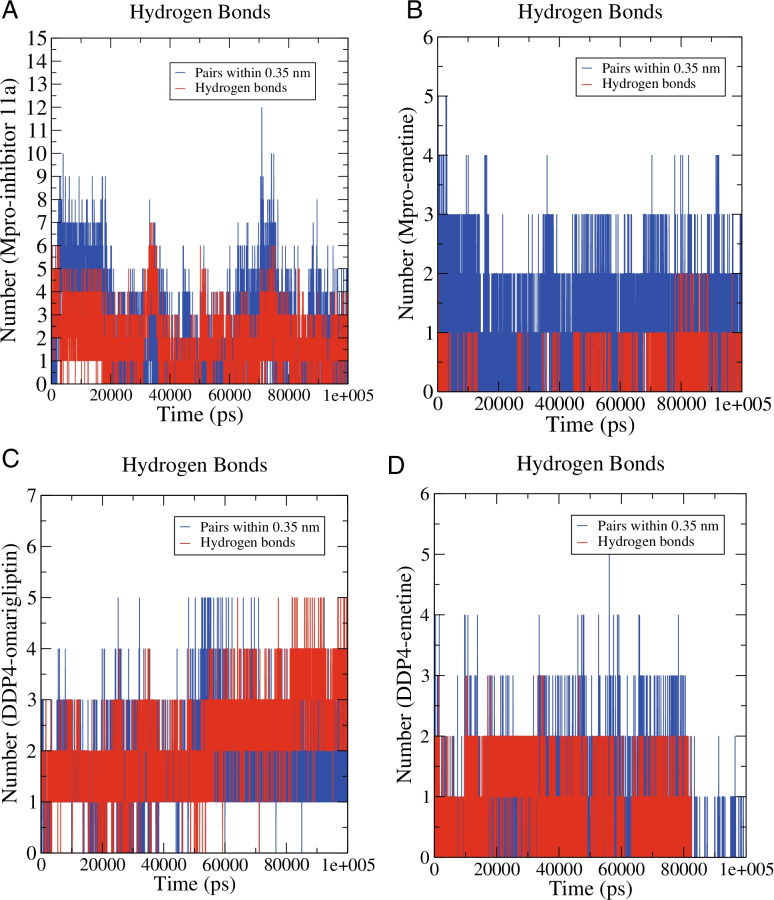
H-bond monitoring reports. **A** Mpro-inhibitor 11a. **B** Mpro-emetine. **C** DDP4-omarigliptin. **D** DDP4-emetine

To explore the binding stability of the proteins and ligands, we evaluated the intermolecular hydrogen bond interactions formed during the 100-ns MD simulation. During the 100 ns of MD simulation, Mpro-inhibitor 11a could form up to 7 hydrogen bonds, and after 20 ns, the number of hydrogen bonds was 1–4 (Fig. 9A). Mpro-emetine could form up to 2 hydrogen bonds and stabilize at forming 1 hydrogen bond after 40 ns (Fig. 9B). DDP4-omarigliptin could form up to 5 hydrogen bonds while 1–3 hydrogen bonds at 0–50 ns and 3–5 hydrogen bonds at 50–100 ns (Fig. 9C). DDP4-emetine could form up to 3 hydrogen bonds and mainly forms 1–2 hydrogen bonds at 0–80 ns (Fig. 9D).

#### Protein-ligand interaction energy

To quantify the strength of the interaction between the proteins and ligands, we calculated the non-bonded interaction energy between them, including Coulomb interactions (Coul) and Van Der Waals interactions. As described in Table 6, compared to inhibitor 11a (Coul − 67.2 ± 6.6 kJ/mol, Vdw − 168.7 ± 10.0 kJ/mol), the interaction energy of emetine with Mpro was higher for Coul and lower for Vdw (Coul − 23.9 ± 2.2 kJ/mol, Vdw − 174.7 ± 2.5 kJ/mol). In addition, both Coul and Vdw were higher in the interaction energy of emetine with DDP4 than that of omarigliptin (omarigliptin: Coul − 86.6 ± 11.0 kJ/mol, Vdw − 133.3 ± 2.7 kJ/mol; emetine: Coul − 27.0 ± 5.9 kJ/mol, Vdw − 108.8 ± 6.4 kJ/mol).

**Table 6:** The non-bonded interaction energy between proteins and ligands (kJ/mol)

Protein-ligand complex	Coul	Vdw	Total interaction energy
Mpro-inhibitor 11a	− 67.2 ± 6.6	− 168.7 ± 10.0	− 235.9 ± 12.0
Mpro-emetine	− 23.9 ± 2.2	− 174.7 ± 2.5	− 198.7 ± 3.3
DDP4-omarigliptin	− 86.6 ± 11.0	− 133.3 ± 2.7	− 219.9 ± 11.3
DDP4-emetine	− 27.0 ± 5.9	− 108.8 ± 6.4	− 135.8 ± 8.7

*Coul* Coulomb interactions, *Vdw* Van der Waals interaction

## Discussion

COVID-19 is a contagious disease with increasing worldwide infection and mortality [2], and specific therapeutic drugs for COVID-19 have not yet been effectively developed [3]. Nowadays, cancer remains one of the major contributors to the worldwide burden of diseases [55], and of various cancers, lung cancer is the dominant cause of cancer incidence and death globally, representing 19.4% of cancer-associated mortalities [56]. In addition, because lung cancer patients often exhibit immune dysfunction [7], they are at higher risk for severe COVID-19 outcomes [57]. Taken together, LUAD patients may be more vulnerable to SARS-CoV-2, which may compromise treatment outcomes and reduce survival in these patients.

In the present study, we initially screened 230 LUAD/COVID-19 DEGs. Then, we constructed a risk score consisting of 7 genes (*BTK*, *CCL20*, *FURIN*, *LDHA*, *TRPA1*, *ZIC5*, and *SDK1*). The model showed that the risk of death in patients with LUAD increased with increasing risk scores. Furthermore, the prognostic prediction model based on this risk score showed satisfactory efficiency for LUAD. The analyses of these 7 gene expression levels between different clinical features suggested that *BTK*, *SDK1*, *CCL20*, *LDHA*, and *ZIC5* may serve as effective biomarkers for screening and characterizing patients with LUAD/COVID-19 at different stages. In short, the 230 LUAD/COVID-19 DEGs are likely to be the potential therapeutic targets. Then, the drug screening results on cMap showed that emetine might have a therapeutic effect on LUAD/COVID-19. Emetine has been previously shown to exert anti-lung cancer effects through multiple pathways. For example, emetine can inhibit the invasion and migration of NSCLC by regulating the ERK and p38 pathways [58]. In addition, low doses of emetine alone or in combination with ganciclovir have shown satisfactory efficacy in treating human cytomegalovirus [59], and some recent studies found that emetine may suppress the replication of SARS-CoV-2 through targeting PLpro or Mpro [17, 18], and the combination of 0.195 μM emetine and 6.25 μM remdesivir was observed to achieve 64.9% inhibition of viral yield in Vero E6 cells [60]. Therefore, we speculated that emetine might exhibit a powerful therapeutic effect in patients with LUAD/COVID-19.

Then, we identified 6 possible targets (*SLC6A4*, *MIF*, *DPP4*, *PRF1*, *SERPING1*, and *IL6*) of emetine against LUAD/COVID-19 by a network pharmacology approach, and the results of functional enrichment analysis of these 6 genes suggested that anti-LUAD/COVID-19 effects of emetine were mediated by antiviral and immunomodulation. Mpro is the most characteristic therapeutic target in SARS-CoV-2, and inhibiting its activity will hinder viral replication [23]. Therefore, we selected Mpro for molecular docking with emetine. The result of redocking showed that the RMSD of the native ligands of Mpro and DDP4 was < 2 Å, which indicated that our molecular docking protocol could effectively predict the binding pattern of emetine and Mpro and DDP4. Previous studies identified THR24, PHE140, ASN142, CYS145, THR26, GLY143, HIS163, HIS164, GLU166, and HIS172 as amino acids located in the Mpro active site [38]. By molecular docking analysis, we found that emetine interacts with GLU-166 and ASN-142 residues of Mpro. The affinity of the native ligand (inhibitor 11a) to Mpro was − 8.3 kcal/mol, while the affinity of emetine to Mpro was − 7.8 kcal/mol, which was much less than − 1.2 kcal/mol, suggesting that emetine may inhibit viral replication by effectively binding to Mpro. In addition, the affinity of emetine with DDP4 and MIF was − 8.7 kcal/mol and − 6.6 kcal/mol, respectively, which were both less than − 1.2 kcal/mol. This result suggested that emetine could bind to DDP4 and MIF, with better affinity to DDP4. Amino acid residues in the active site of DDP4 include HIS740, SER630, ARG125, GLU205, GLU206, TYR547, PHE347, SER209, TYR585, and ARG348 [34, 40], and GLN553 was associated with increased inhibitory potency of DDP4 inhibitors [61]. We found that emetine may interact with TYR547, TYR585, and GLN553.

Next, we performed 100-ns MD simulations of the protein-ligand complexes obtained by molecular docking and compared them with the corresponding co-crystal inhibitors in each case. The average RMSD of the protein in the complex systems of emetine bound to Mpro and DDP4 was around 2 Å, while the average RMSD of emetine was also around 2 Å. This suggested that emetine was tightly bound to the pocket of Mpro and DDP4 and therefore has conformational stability. The RMSF and radii of gyration of Mpro and DDP4 confirmed the conformational stability upon binding to emetine. H-bond monitoring showed that emetine could form relatively stable hydrogen bonds with Mpro and DDP4, although the number of hydrogen bonds was reasonably smaller than that of the native ligands. In addition, the total interaction energy of emetine with Mpro and DDP4 was − 198.7 ± 3.3 kJ/mol and − 135.8 ± 8.7 kJ/mol, respectively, which was higher than that of native ligands. This suggested that emetine was not as tightly bound to the protein as native ligands. However, this result was enough to further confirm the good binding ability of emetine with Mpro and DDP4.

**Fig. 10:**
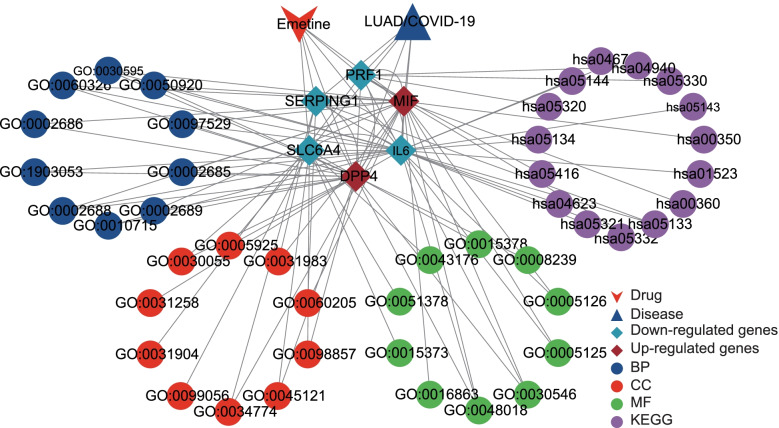
The network of emetine against LUAD/COVID-19 targets and the interactions of related pathways

The DGE analysis indicated that DDP4 and MIF were upregulated genes, and MIF was related to the survival of the patients with LUAD. DPP4 is a serine protease by which the N-terminal proline or alanine of many peptides can be hydrolyzed [62]. The role of DPP4 has been extensively studied in several cancers. For example, the tumor suppressor p53 can restrict colorectal cancer cell ferroptosis by inhibiting the activity of DPP4 [63], and inhibition of DDP4 activity enhances lymphocyte transportation and improves tumor immunotherapy [64]. Interestingly, DDP4 has been little studied in lung cancer. In addition, DPP4 is a cellular receptor for Middle East respiratory syndrome coronavirus [65]. A recent study based on a bioinformatics approach of protein crystal structure predicted that DDP4 could be a potential binding target for SARS-CoV-2 spike protein, which can assist the virus in entering the cell [66]. These studies provided a supportive explanation for exploring the mechanism of emetine anti-LUAD/COVID-19 in our research. We hypothesized that, on the one hand, emetine enhanced lymphocyte trafficking and improved naturally occurring tumor immunity and immunotherapy by inhibiting the activity of DDP4. On the other hand, emetine may reduce SARS-CoV-2 entry into cells by binding to DDP4 and inhibit SARS-CoV-2 replication by binding to Mpro. MIF is a cytokine closely associated with cancer and functions as a promoter in inflammation, and inhibition of MIF can suppress cancer cell proliferation [67]. Also, a study confirmed that proteolysis targeting chimera designed based on MIF tautomerase active site exhibited excellent anti-proliferative activity in lung cancer cells [68]. Moreover, exposure to SARS-CoV-2 spike protein combined with hypoxia enhanced MIF production [69]. Also, MIF can induce lung inflammatory cytokines in the COVID-19-induced inflammatory reaction [70]. The results suggested that the intersecting genes were likely to be effective therapeutic targets of emetine against LUAD/COVID-19 (Fig. 10). Taken together, we believe that emetine may improve the curative effect of antivirals and immunotherapy for anti-COVID-19 or anti-LUAD/COVID-19, which requires further validation.

However, our study still has some limitations. Firstly, the risk score was obtained by retrospective data analysis, and prospective studies are needed to validate the model. Secondly, the mechanism exploration was done based on network pharmacology, molecular docking, and MD simulations, which still needs to be validated by animal experiments or even clinical trials.

## Conclusions

In summary, by bioinformatic analyses, we firstly constructed a model for predicting the prognosis of LUAD patients. We then screened emetine as a potential drug for anti-LUAD/COVID-19 and highlighted antiviral and immunomodulation as the critical pathways for emetine against LUAD/COVID-19. In addition, the results of network pharmacology, molecular docking, and MD simulations indicated that emetine might reduce SARS-CoV-2 entry and inhibit SARS-CoV-2 replication by binding DDP4 and SARS-CoV-2 Mpro, and enhance tumor immunity by inhibiting DDP4 activity.

## Supplementary Information

**Additional file 1.** Genes related to COVID-19.

**Additional file 2.** Intersection genes of LUAD DEGs and COVID-19-related genes.

**Additional file 3.** Univariate Cox proportional hazards regression analysis of the intersecting genes of COVID-19 and LUAD.

**Additional file 4.** GO enrichment analysis of the targets of emetine against LUAD/COVID-19.

**Additional file 5.** KEGG enrichment analysis of the emetine’s anti-LUAD/COVID-19 targets.

**Additional file 6.** Molecular docking of emetine with MIF.

**Additional file 7.** Radii of gyration of Mpro and DDP4.

**Additional file 8.** The redocking files.

## Data Availability

The datasets generated and/or analyzed during the current study are available in the figshare repository, 10.6084/m9.figshare.19126205

## Abbreviations

COVID-19: Coronavirus disease 19
SARS-CoV-2: Severe acute respiratory syndrome coronavirus 2
LUAD: Lung adenocarcinoma
TCGA: The Cancer Genome Atlas
DEG: Differentially expressed gene
NSCLC: Non-small cell lung cancer
PLpro: Papain-like protease
Mpro: Main protease
GO: Gene Ontology
KEGG: Kyoto Encyclopedia of Genes and Genomes
RMSD: Root mean square deviation
RMSF: Root mean square fluctuation
MD: Molecular dynamics
H-bond: Hydrogen bond
