# Correction: Exploring the potential mechanism of emetine against coronavirus disease 2019 combined with lung adenocarcinoma: bioinformatics and molecular simulation analyses

**DOI:** 10.1186/s12885-022-09836-2

**Published:** 2022-07-27

**Authors:** Kun Zhang, Ke Wang, Chaoguo Zhang, Xiuli Teng, Dan Li, Mingwei Chen

**Affiliations:** Department of Respiratory and Critical Care Medicine, First Afliated Hospital of Xi’an Jiaotong University, No. 277 Yanta West Road, Xi’an, 710061 Shaanxi Province China

**Correction: BMC Cancer 22, 687 (2022)**

**https://doi.org/10.1186/s12885-022-09763-2**

Following publication of the original article [1], the authors identified a production error. Figure 8 was not correctly processed by the publisher. The correct version of figure 8 is supplied in this correction article.

**Fig. 8:**
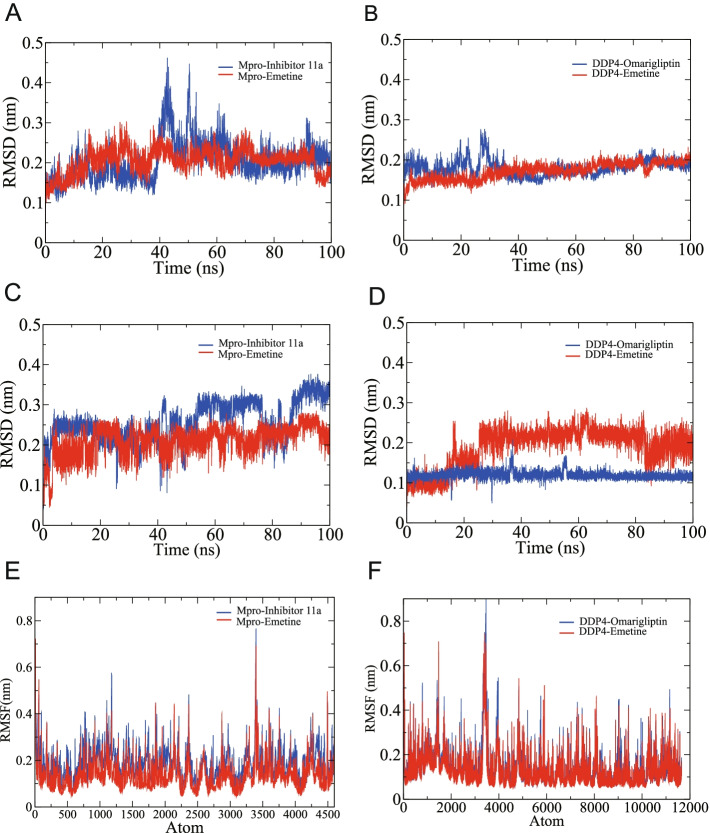
RMSD and RMSF of the SARS-CoV-2 Mpro complexes and DDP4 complexes. **A** The RMSD of Mpro. **B** The RMSD of DDP4. **C** The RMSD of inhibitor 11a and emetine. **D** The RMSD of omarigliptin and emetine. **E** The RMSF of Mpro. **F** The RMSF of DDP4
