# Supplementary figures and images for: Exploring the potential mechanism of emetine against coronavirus disease 2019 combined with lung adenocarcinoma: bioinformatics and molecular simulation analyses
Source: BMC Cancer. 2022 Jun 22;22:687. doi: 10.1186/s12885-022-09763-2 (PMC9214478; doi:10.1186/s12885-022-09763-2)

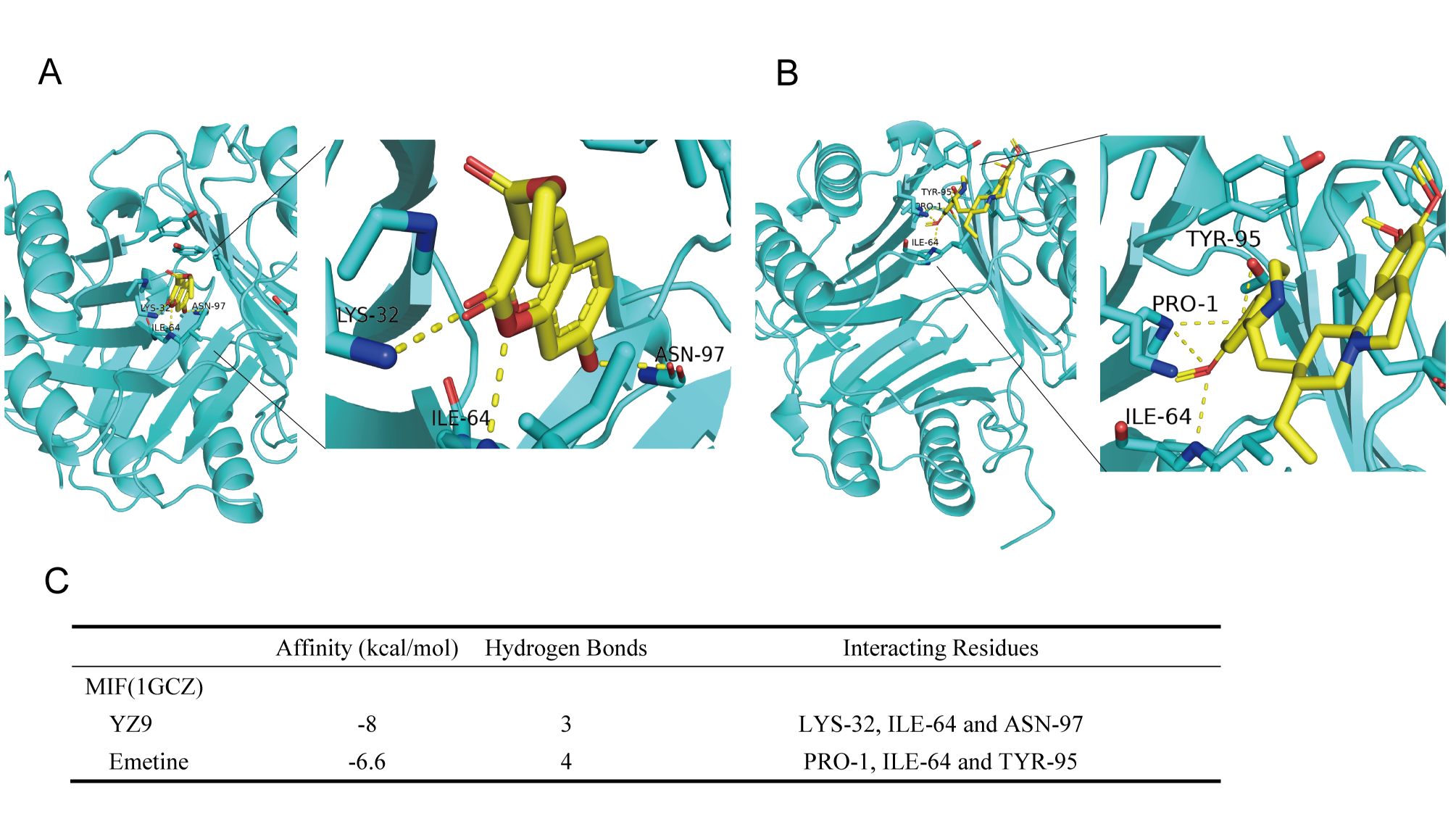

Supplement: Supplementary file 6 — Additional file 6. Molecular docking of emetine with MIF. [file 12885_2022_9763_MOESM6_ESM.tif]

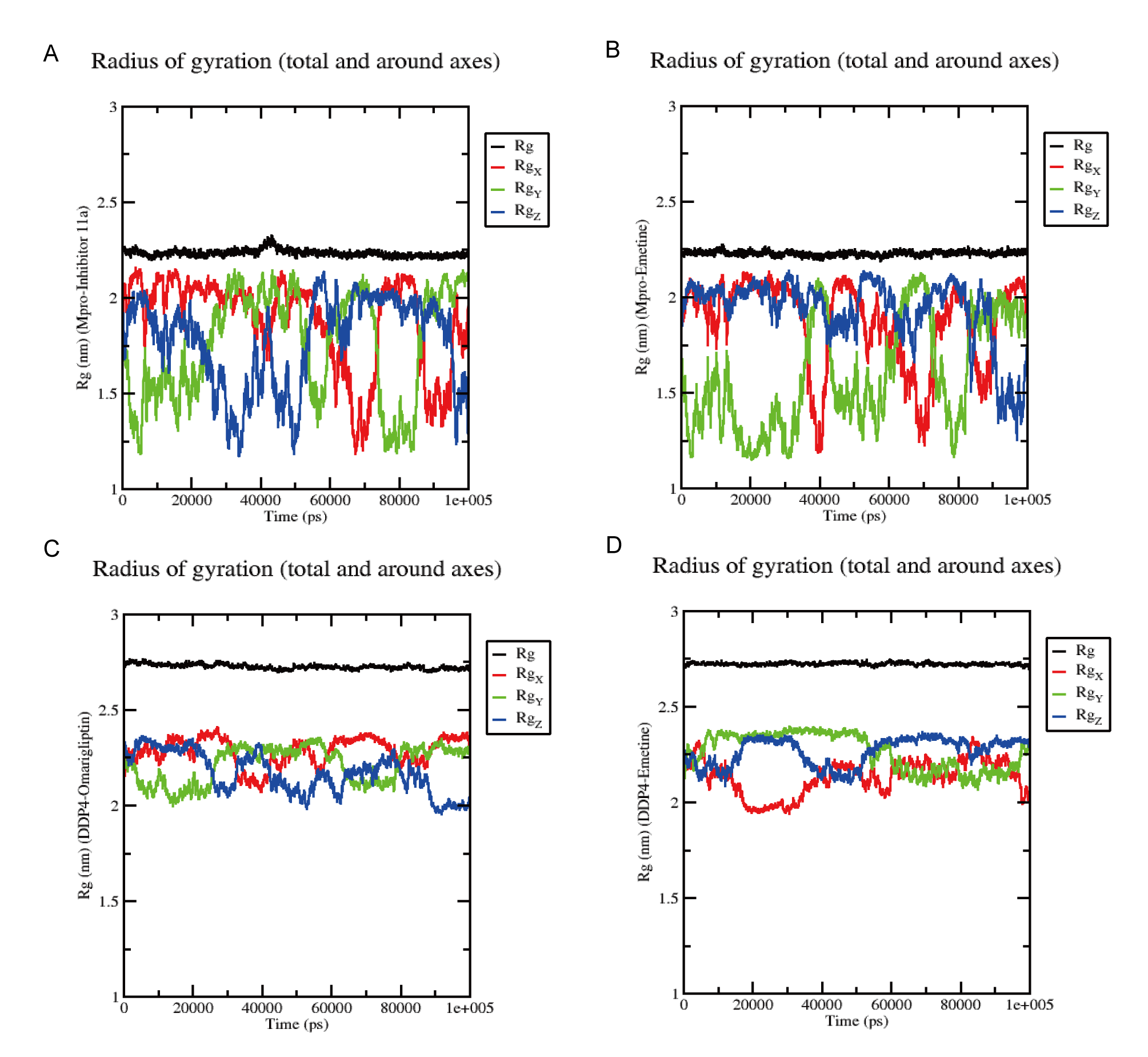

Supplement: Supplementary file 7 — Additional file 7. Radii of gyration of Mpro and DDP4. [file 12885_2022_9763_MOESM7_ESM.tif]

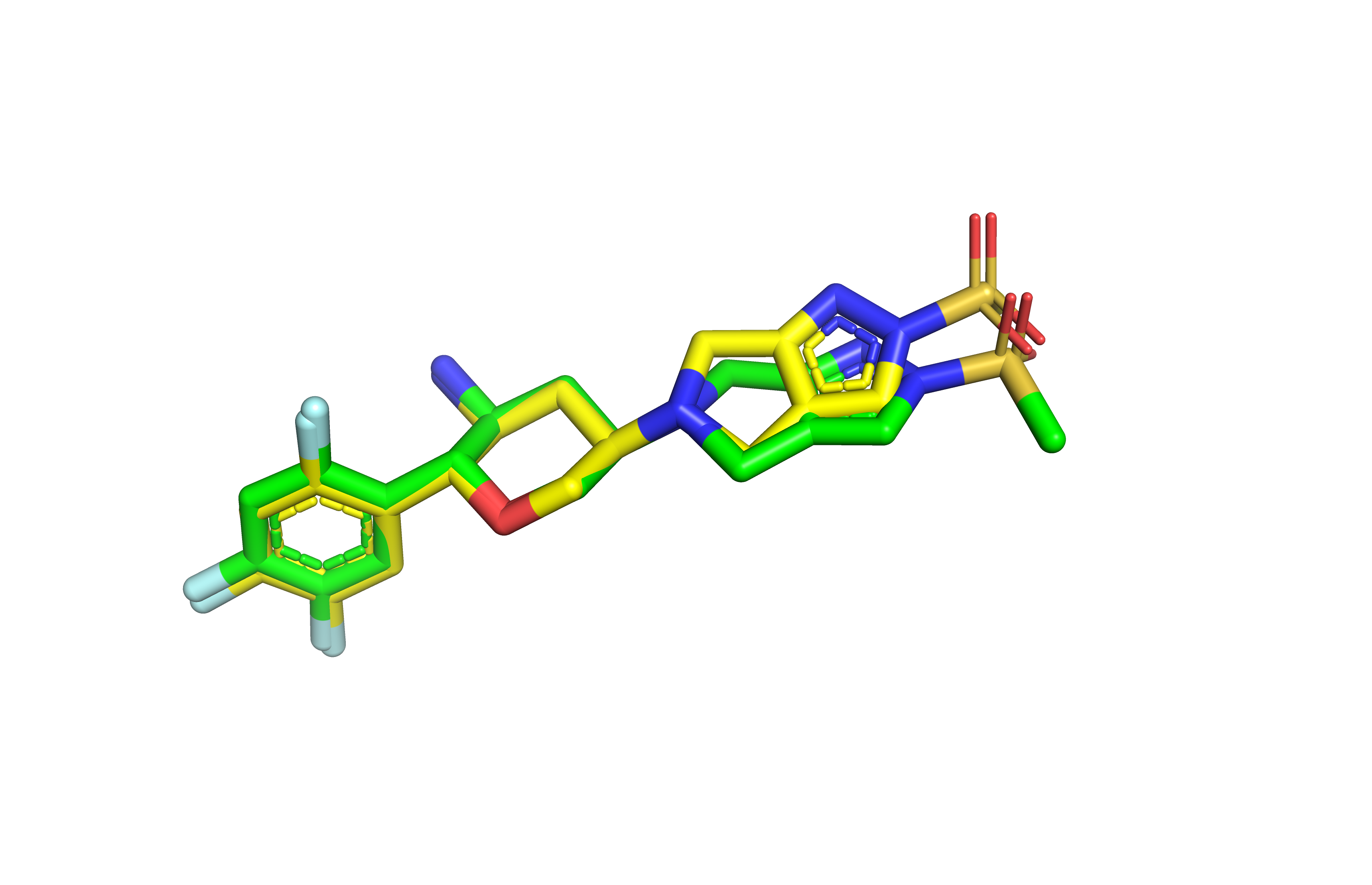

Supplement: Supplementary file 8 — Additional file 8. The redocking files. [file 12885_2022_9763_MOESM8_ESM.zip › Additional file 8/DDP4-Omarigliptin/Omarigliptin-RMSD.png]

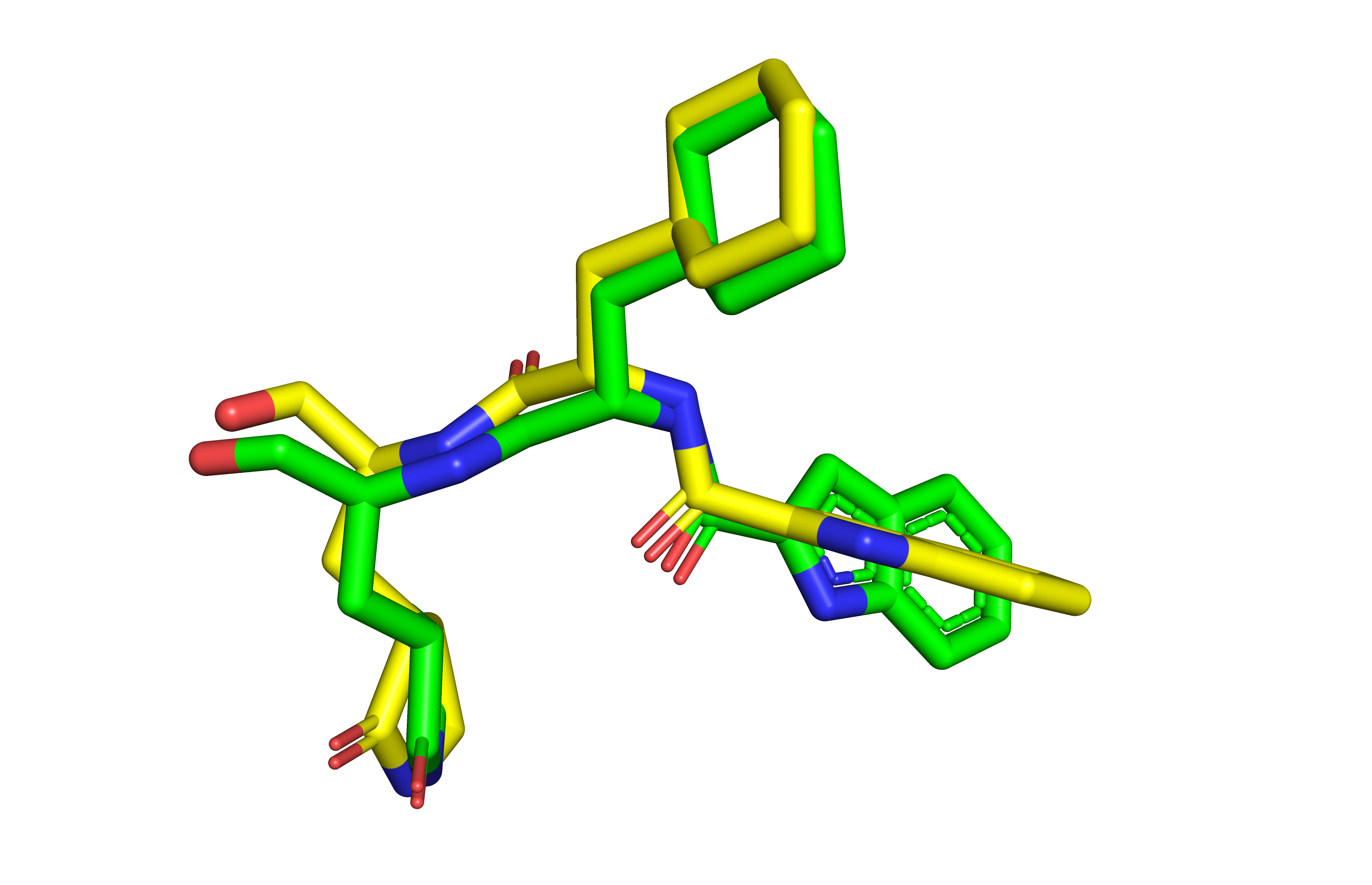

Supplement: Supplementary file 8 — Additional file 8. The redocking files. [file 12885_2022_9763_MOESM8_ESM.zip › Additional file 8/Mpro-Inhibitor11a/Inhibitor11a_rmsd.png]
